# Vector-Borne Tularemia: A Re-Emerging Cause of Cervical Lymphadenopathy

**DOI:** 10.3390/tropicalmed7080189

**Published:** 2022-08-16

**Authors:** Kaja Troha, Nina Božanić Urbančič, Miša Korva, Tatjana Avšič-Županc, Saba Battelino, Domen Vozel

**Affiliations:** 1 Department of Otorhinolaryngology and Cervicofacial Surgery, University Medical Centre Ljubljana, 1000 Ljubljana, Slovenia; 2 Faculty of Medicine, University of Ljubljana, 1000 Ljubljana, Slovenia; 3 Institute of Microbiology and Immunology, Faculty of Medicine, University of Ljubljana, 1000 Ljubljana, Slovenia

**Keywords:** ticks, lymph nodes, vector-borne diseases, lymph node excision, serology, bioterrorism

## Abstract

Tularemia is a zoonosis caused by the highly invasive bacterium *Francisella tularensis*. It is transmitted to humans by direct contact with infected animals or by vectors, such as ticks, mosquitos, and flies. Even though it is well-known as a tick-borne disease, it is usually not immediately recognised after a tick bite. In Slovenia, tularemia is rare, with 1–3 cases reported annually; however, the incidence seems to be increasing. Ulceroglandular tularemia is one of its most common forms, with cervical colliquative lymphadenopathy as a frequent manifestation. The diagnosis of tularemia largely relies on epidemiological information, clinical examination, imaging, and molecular studies. Physicians should consider this disease a differential diagnosis for a neck mass, especially after a tick bite, as its management significantly differs from that of other causes. Tularemia-associated lymphadenitis is treated with antibiotics and surgical drainage of the colliquated lymph nodes. Additionally, tularemia should be noted for its potential use in bioterrorism on behalf of the causative agents’ low infectious dose, possible aerosol formation, no effective vaccine at disposal, and the ability to produce severe disease. This article reviews the recent literature on tularemia and presents a case of an adult male with tick-borne cervical ulceroglandular tularemia.

## 1. Introduction

Tularemia is a well-known, yet rare zoonotic disease caused by *Francisella tularensis* (*F. tularensis*). The bacterium was first isolated in 1911 by McCoy and Chapin from infected squirrels in Tulare County, California. It was named after the place of discovery and after the researcher Edward Francis, who first described the disease and conducted epidemiological research [1,2].

The epidemiology of the disease has changed significantly in recent decades, and outbreaks in our region are likely. Therefore, physicians should not overlook tularemia after tick bites, although these are usually related to other, more common diseases (e.g., borreliosis) [3,4]. It is also vital to consider tularemia as a possible differential in a patient presenting with a neck mass because of its specific treatment.

Additionally, *F. tularensis* should be noted for its potential use in bioterrorism on behalf of the causative agents’ low infectious dose, possible aerosol formation, no effective vaccine at disposal, and the ability to produce a severe disease [5]. According to the U.S. Department of Health and Human Services, it is classified as a List A agent of the most severe concern of bioterrorism use [6,7].

The objective of this paper is twofold: (1) to review the current literature and revisit the characteristics of tularemia and (2) to describe and discuss the case of an adult male with an ulceroglandular form of tularemia in cervical lymph nodes after a tick bite.

## 2. Pathogenesis of Tularemia

*F. tularensis* is a gram-negative, aerobic, facultative intracellular bacterium. Taxonomically, it is divided into four known subspecies: *F. tularensis* subsp. *tularensis*, *F. tularensis* subsp. *holarctica*, *F. tularensis* subsp. *Mediasiatica,* and *F. tularensis* subsp. *novicida*. Earlier publications considered *F. novicida* and *F. tularensis* as a separate species. *F. novicida* differs from other subtypes in its lessened ability of tissue invasion and tissue damage in mammals [8]. It is also not a part of the select agent list of the United States and does not require as excessive laboratory safety regulations as other subtypes [9]. The re-classification of subspecies, however, remains debatable [10,11].

Subsp. *tularensis* and *holarctica*, previously called type A and B, respectively, are the most relevant subtypes for clinical practice [12,13]. Subsp. *tularensis* is almost exclusively present in Northern America and causes a more severe form of the disease. It is particularly virulent, with an infectious dose of <10 colony forming units (CFUs) [1,14]. The type A strains are further separated into subtypes A.I and A.II, with A.I being the most virulent [15]. In cases of pulmonary infections with subsp. *tularensis* without treatment, the mortality of up to 60% is described [16]. The less virulent type subsp. *holarctica* with the infectious dose of 100–1000 CFUs causes the majority of infections in Europe [7,17]. The infection results in a milder, sometimes even subclinical disease [12,18,19]. Both pathogens can persist in the environment for weeks or even months [20].

The bacteria enter the body through minor skin or mucosa wounds. The capsule of *F. tularensis* is an essential virulence factor, enabling it to dodge polymorphonuclear neutrophil destruction. As it enters the bloodstream, it is phagocyted by circulating monocytes and macrophages of the reticuloendothelial system, where it can survive as an intracellular parasite. As a result, granulomatous lesions can arise in the affected organs [14,21]. Immunity after the infection is usually life-long, although reinfections have been described in the literature [22].

According to Svensson et al. (2005), the genome of *F. tularensis* has recently shown an evolutionary change [23]. Additionally, a few cases of previously uncommon human infections with *F. tularensis novicida*-like strains have been described recently in tropical Australia and Thailand [24,25]. The genome of *F. tularensis* has also been researched in our geographical space. Glinšek Biškup et al. (2021) analysed the molecular diversity of *F. tularensis* in tularemia confirmed cases in Slovenia. Subsp. *holarctica* was the causative agent in all of the studied patients [26].

## 3. Transmission of Tularemia

*F. tularensis* was isolated from more than 100 wild animal species, domestic animals, arthropods, birds, and fish. The main reservoirs are wild mammals, such as rabbits, squirrels, and beavers. People working with these animals (e.g., hunters, butchers, and furriers) are more exposed to the infection [13,27,28].

Tularemia is transmitted to humans by direct contact with an infected animal (e.g., rabbits, beavers, hares, and rodents), most commonly when working with its meat and skin, or by arthropod bites (e.g., ticks, mosquitoes, flies, and lice). These have been previously feasting on an infected animal or water source. Other possible transmission routes are ingesting contaminated water or food and inhaling aerosolised bacteria, for instance, in hay or while handling the pathogens in laboratories [18,29]. Grass mowing, hay stacking, and other activities with possible machine driving over infected animals or their carcasses are hazardous for aerosol formation [27,30]. Tularemia has not been reported to transmit directly from human to human; hence, contact isolation of infected persons is not necessary [31].

Transmission of tularemia usually occurs from May to August during hunting season. In the USA, ticks, deer flies, and rabbits are the most common sources of infection, whereas in Northern Europe, rodents, mosquitos, and the tick *Dermacentor reticulatus* are the most common disease agent carriers [32,33].

## 4. Tularemia as a Tick-Borne Disease

Despite the well-established transmission route by tick bites, tularemia is rarely immediately recognised as a tick-borne disease [16]. Ticks were discovered as vectors in 1924 [34]. Tick-borne tularemia cases are reported in almost all endemic areas [35]. More than 85% of all tularemia confirmed cases in the 1960s were associated with tick bites in the United States, where still around half of the tularemia infections are related to tick bite exposure. From 2004 to 2016, 2.102 tick-borne tularemia cases were reported in the USA [36,37]. In Europe, recent reports have changed the previously understated importance of tick-borne transmission of tularemia [38]. According to Maurin et al. (2011), 11% of tularemia cases are tick-borne in France, while Guycova et al. (2010) described 12.8% of tick-borne tularemia confirmed cases in Slovakia [39,40].

Epidemiological data in a study by Rojko et al. (2016) regarding tularemia cases between 2012 and 2013 in Slovenia revealed tick-borne infections in 50% of patients. Furthermore, *Ixodes ricinus* was found to be the most prevalent species of vectors in Slovenia. In contrast, *Dermacentor reticulatus* was characterised only in the northeastern part of the country, where most tularemia cases were reported before 2012 [3].

## 5. Epidemiology of Tularemia

Even though tularemia is a well-studied disease, with its causative bacterium isolated in many countries, its occurrence overall is rare. However, sporadic cases and outbreaks are reported around the globe, predominantly in the northern hemisphere between 30° and 70° latitude [41]. Approximately 800 cases are reported annually in Europe. The disease is thought to be endemic in Sweden and Finland, the countries with the most cases reported in Europe. Mosquito bites are this area’s most common transmission route [42]. Kosovo likewise reports one of the highest incidence rates of tularemia in Europe. The highest incidence in this country was reported in 2010, with 11.26 cases per 100,000 inhabitants [43,44]. In addition to Kosovo (1999, 2000, and 2003), the most significant outbreaks have been reported in Turkey (2005–2007) and Spain (1997, 1998, and 2007) [41]. In Europe, tularemia has not been reported in Iceland, Ireland, or the United Kingdom [45].

In the past decades, the epidemiology of the disease has changed, with several outbreaks reported in areas previously considered non-endemic. Tularemia is regarded as a locally emerging or re-emerging disease in Europe [4]. It has recently expanded its geographical range and included host animals previously not linked to tularemia, such as the red fox, the wild boar, and the raccoon dog [46,47]. The European Food Safety Authority (EFSA) and the European Center for Disease and Prevention Control (ECDC) have reported increased animal reservoirs [48]. The dynamics in epidemiology seem to have been the opposite overseas. In the USA, in the 1950s, almost 1000 cases were reported annually, whereas in 2019, a little over 270 cases were reported to the CDC [36].

In Slovenia, the disease is rare, with 1–3 cases reported annually [30,31], but the numbers have risen in the past decades [44]. From 1990 to 2020, 42 cases of tularemia were reported. Before 2012, only eight sporadic cases had been reported over the past ten years. Half of the cases occurred in the northeastern part of Slovenia. Recently, cases have been described in other areas of the country. The first clinical cases outside of the northeastern region were reported in 2012. In 2012 and 2013, a cluster of six cases occurred in the central part of Slovenia [30,49]. Epidemiological data by Rojko et al. (2016) report 31 patients with clinical diagnosis of tularemia treated in Slovenia from 2004 to 2018 [3].

Additionally, in 2021, there was an outbreak of tularemia mainly in the western part of Slovenia, with more than 35 cases confirmed, but, in other parts of Slovenia, an increase of sporadic cases was also observed [4]. To the best of our knowledge, there is no description of cervical ulceroglandular tularemia in our country yet.

## 6. Clinical Presentation of Tularemia

In most cases, tularemia presents as an acute febrile illness with a high fever, lymphadenopathy, and ulcerative lesions on inoculation sites. The symptoms occur after 3–4 days (1–21 days) of incubation with sudden febrile illness, shivers, malaise, fatigue, and headache. The course of the disease depends on the causative agent’s transmission route, entry site, and degree of virulence [16].

Ulceroglandular, oculoglandular, oropharyngeal, pulmonary, and typhoid tularemia are the most common forms of the disease [1,13]. However, a more straightforward division into ulceroglandular and typhoid forms is suggested in recent literature [16,27,50,51]

In 80% of cases, the bacteria cause the ulceroglandular form of the disease, which usually presents with shivers, fever, headache, cough, myalgia, abdominal pain, vomiting, and diarrhoea. After the initial symptoms, a painful papule occurs over the body part of the microorganism’s entry point. The papule usually necrotises and evolves into a painful ulcer with an elevated border. Without antibiotic treatment, it heals with a keloid and persists for an extended period. Lymph node enlargement around the inoculation site progresses to colliquation, which is palpable as fluctuation. Untreated, the infection lasts an average of 32 days, although cases of a disease with the less virulent type B persisting for only a few days are possible [28,50].

The glandular type of the disease is equal to the ulceroglandular one, with no signs or symptoms manifesting on the skin [52]. However, supposing the bacteria enter the body through the eyes, the oculoglandular form of the disease can develop, such as severe conjunctivitis, yellow ulcers on the conjunctiva, and neck lymphadenopathy. These manifest as Parinaud’s oculoglandular syndrome, which can, in addition to *F. tularensis,* be caused by other microorganisms (e.g., *Bartonella*, *Rickettsia*, and Epstein–Barr virus) [52].

Uncommonly, the disease presents as pharyngeal tularemia usually contracted after drinking contaminated water. Odynophagia, exudative tonsillopharyngitis, and severe neck lymph node swelling are typically seen in this form of the disease. Typhoid tularemia is another form of the disease, seen as an acute febrile illness without skin manifestations or lymphadenopathy. Systemic symptoms are pronounced—malaise, fever, pain in the throat, cough, malaise, vomiting, diarrhoea, sometimes hypotension, hepatomegaly, and splenomegaly. The most severe form of the disease is pulmonary tularemia, presenting as severe pneumonia, and is deadly in 60% if left untreated. Chest X-ray typically reveals enlarged hilus lymph nodes [27,53,54].

According to the reviewed literature, up to 45% of patients with tularemia present with otorhinolaryngological symptoms (such as cervical lymphadenopathy), which are the usual manifestations of ulceroglandular or glandular forms of the disease [6,39,55]. When the transmission is associated with a tick bite, the site of the bite determines the location of affected lymph nodes. Generalised lymphadenopathy is rarely seen [56]. Cervical lymphadenopathy is widespread in children, while most adult cases are described with inguinal lymphadenopathy [57]. Hanke et al. (2009) reported a case of ulceroglandular tularaemia of possibly one of the youngest patients, a 16-month-old toddler. The disease developed after a mosquito bite with fever, ulceration, and preauricular lymphadenopathy [58]. Pediatric tularemia has been reviewed by Kukla et al. (2021) with two case reports of pediatric patients with tick-borne tularemia with lymphadenitis, concluding that tick-borne tularemia was important as a differential diagnosis of lymphadenitis even in children under three years of age [59].

Several all-age groups of ulceroglandular tularemia cases with clinical presentation as lymphadenitis have been reported. Wills et al. (1982) reviewed eighty-one cases of tularemia in a study from 1970 to 1980. Head and neck findings were described in fourteen cases (17%). Twelve patients had cervical lymphadenitis, and six reported tick-bites in recent history [60]. Atmaca et al. (2008) conducted a retrospective study with 145 patients in 5 years with neck mass without known primary malignancy; twenty-six patients (17.9%) were diagnosed with tularemia. This study showed how an outbreak in Turkey in 2004 changed the management of patients presenting with neck masses, after which serologic testing for tularemia was routinely performed in inflammatory neck mass investigations [61]. Strehl et al. (2014) report five adult cases of tularemia-related lymphadenitis from 2007 to 2013. Three patients had cervical lymph node enlargement, two of which formed an abscess in the affected lymph node area [16]. Borde et al. (2017) report five cases of tick-borne tularemia, where only one was described as cervical lymphadenitis [62].

An essential study for our geographical space is by Rojko et al. (2016), describing a cluster of six ulceroglandular tularemia cases in Slovenia. Three of six adult patients reported a tick bite before developing symptoms of swollen regional lymph nodes, high fever, chills, headache, general malaise, sore throat, nausea, vomiting, and cough. One of the patients had cervical (infra and supraclavicular) lymphadenitis. The other two suffered from inguinal lymphadenopathy. Erythema was observed at the location of the primary lesion as well. The patient with cervical lymphadenopathy experienced six days of fever with headache, nausea, and vomiting and was diagnosed 17 days after the onset of the disease [3].

## 7. Complications of Tularemia

There are possible local and systemic complications of tularemia, such as disseminated intravascular coagulation, renal failure, hepatitis, meningitis, endocarditis, myocarditis, peritonsillar abscess, and spleen rupture and osteomyelitis [35,41]. The severe form of the disease, the typhoid type, can ultimately lead to septicemia, pneumonia, endocarditis, meningitis, and rhabdomyolysis [28,50]. Despite their extremely rare occurrence, prompt diagnosis of the disease is crucial. Equally noteworthy, local complications can either significantly worsen patients’ quality of life (i.e., chronic lymphadenopathy and spontaneous ulceration requiring wound care for weeks) or even result in life-threatening abscess formation. Cervical lymphadenopathy can spread lymphatically with regional suppurative lymphadenopathy and can, in the most severe cases, progress into deep neck space infection, mediastinitis, and sepsis [51,63].

As described in the literature review, suppuration of the involved lymph nodes is the most common complication of ulceroglandular tularemia. Suppuration has been reported to occur in 30% of tularemia-associated lymphadenitis cases [64]. Nemmour et al. (2019) described a case of a 5-year-old Swiss boy with a parapharyngeal abscess as a complication of oropharyngeal tularemia presenting with fever, sore throat, and unilateral cervical lymphadenopathy. The transmission route was by contact with a dead mouse [65]. To a similar extent, complications have been described in adults as well. An American study by Alsan and Lin (2010) reported a glandular form of tularemia. The infection was obtained from an undefined arthropod vector and presented as a superficial cervical abscess [66].

Additionally, a study by Kaeppler et al. (2020) shows a case of tick-borne tularemia with lymphadenopathy resulting in endocarditis. Fever and the presence of a prosthetic cardiac valve raised suspicion of infective endocarditis in their patient [67]. In a study of 13 tularemia cases by Frischknecht et al. (2019), a case of myocarditis secondary to *F. tularensis* is described [68]. In type A tularemia, pneumonia occurs in up to 30% of ulceroglandular cases, whereas it only rarely occurs as a result of type B disease [69]. The longer the delay in diagnosis, the higher the incidence of such complications, as seen in an outbreak in Spain in 2001 with a significant delay in diagnosis when 5 of 142 patients with type B tularemia developed pneumonia [70].

## 8. Diagnosing Tularemia

As the symptoms of tularemia are unspecific and the incidence in the general population is low, tularemia is rarely recognised by primary care physicians. This contributes to delays in diagnosis for several weeks [3].

The diagnosis of lymphadenitis secondary to *F. tularensis* relies on suspicion raised by patient history and epidemiologic information, thorough clinical examination, including upper aerodigestive tract flexible endoscopy in otorhinolaryngological practice, imaging, serology, bacterial DNA amplification by PCR, and/or bacterium culture isolation.

A zoonosis should be suspected when a recent history of exposure to tularemia-related animals such as rabbits, ticks, and rodents is elicited. For that reason, epidemiological information is crucial in a patient with lymphadenopathy. During the physical examination, physicians should seek ulcerative lesions in children, especially those hidden in the scalp [59]. In addition, information about other constitutional symptoms such as fever, myalgia, and pulmonary or gastrointestinal symptoms should be obtained. Microbological isolation of the causative bacterium can be performed from a skin ulcer, lymph node drainage samples, or blood. Owing to the high biosafety risk, these procedures can only be performed in high-containment laboratories with biosafety level 3. Therefore, the assigned laboratory must be warned of suspicion in advance [6,71].

According to recent literature, bacteria isolated from clinical samples (e.g., skin ulcers, lymph node biopsy, and blood) is obtained in less than 10% of patients, which can be attributed to prior antibiotic treatment. The empirical antibiotic treatment of neck lymphadenopathy is one of the factors prolonging the time to diagnosis. Additionally, the prolonged growth of bacteria may mislead physicians into a false negative result [72,73]. Thus, safer and quicker methods are usually applied. Real-time PCR is widely used to confirm the presence of the causative organism DNA in lymph node aspirates or ulcer swabs, even in the early stages of the disease [74]. A limitation of PCR in diagnosing tularemia is its relatively low sensitivity, which has been reported to be around 50% to 72%. Serology has proven to be the most reliable method. In most cases, serology methods often contribute to the final confirmation of the disease. Antibodies are formed 14–21 days after the beginning of the disease’s clinical manifestation, which physicians should keep in mind to avoid premature exclusion of this disease. The diagnosis is confirmed when a single titre of 1:160 (agglutination assay or immunofluorescence assay) or greater or a fourfold increase in titre is found. Antibody titres peak at 3–4 weeks after the development of clinical symptoms, and then titres decrease, while elevated residual titres may persist for months to years [28,39,72]. Serological tests are considered highly specific, but a serological cross-reaction with *Brucella* species is possible [41].

Routine serum chemistry and peripheral blood count may be normal in cases of tularemia. Either the studies can reveal leukocytosis with moderately elevated CRP. A differential white blood count can provide diagnostic clues in cases of lymphadenitis of unknown origin, as an increase in T-cells is indicative of intracellular pathogen infection, such as *F. tularensis* [14,41].

**Figure 1:**
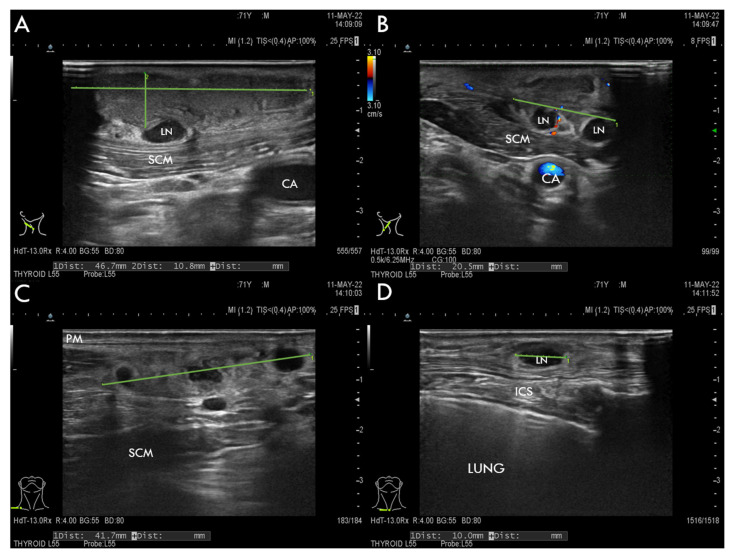
Ultrasonographic neck evaluation of the patient with ulceroglandular tularemia. (**A**) The hypoechoic area of 5 cm × 1 cm × 2 cm (green lines) corresponds to inflammatory changes in subcutaneous fat involving the platysma muscle (PM) above the sternocleidomastoid muscle (SCM) with abnormal adherent lymph node (LN); (**B**) Doppler ultrasonography shows the absence of blood flow in liquid-filled structures above SCM, which corresponds to partly colliquated abnormal lymph nodes (green line) and a non-compressible arterial flow under SCM, which corresponds to the carotid artery (CA); (**C**) a conglomerate of abnormal partially colliquated lymph nodes (green line) above SCM; (**D**) a conglomerate of abnormal 40 mm × 7 mm large supraclavicular lymph nodes overlying the intercostal space (ICS). Panels (**A**–**C**) anatomically correspond to a blue and red arrow in Figure 2. Panel (**D**) anatomically corresponds to the black arrow in Figure 2.

Different imaging studies are essential in establishing a diagnosis, especially with patients presenting with a neck mass. Ultrasound is the most commonly used method to evaluate a persistent neck mass initially. To further assess the extent of the disease and more specific characteristics, CT or MRI are performed [75,76]. In cases of tularemia, sonography usually demonstrates colliquative suppurative lymphadenopathy (Figure 1). Important ultrasound characteristics of tularemia are colliquation and necrosis signs [16,76]. These distinguish tularemia-associated lymphadenitis from a widespread and much public-attention-receiving tick-borne disease in our geographical field—Lyme disease. In around 1% of patients, Lyme disease presents with borrelial lymphocytoma. This is a cutaneous B cell pseudolymphoma, a usually solitary, soft, and non-tender bluish-red nodule or swelling, possibly mimicking suppurated enlarged lymph nodes. Abscess formation and colliquation are not typical characteristics in borrelial lymphocytoma [77,78].

An ultrasound-guided needle biopsy can facilitate the diagnosis of tularemia before surgical excision of the lymph nodes (diagnostic or therapeutic). Histopathological examination of lymph node tissue typically reveals non-specific suppurative inflammation with central necrosis granulomas. Strehl et al. (2014) describe a case of lymphadenitis in tularemia without the classic central necrosis, resembling the sarcoidosis histopathological presentation. According to the authors, this could be seen in the early phases of tularemic lymphadenitis. Therefore, tularemia should not be excluded in cases of absence of central necrotic colliquated lymph node presentation, which is a typical finding in this entity [16].

## 9. Differential Diagnosis of Cervical Lymphadenopathy after a Tick Bite

Lymphadenopathy is one of the leading presenting symptoms in tularemia. Cervical lymph nodes are commonly affected, depending on the entry site and transmission route, especially in oropharyngeal or ulceroglandular forms [27]. Tularemia is rarely considered in patients presenting with a neck mass without epidemiologic data. The work-up of cervical lymphadenopathy is challenging in patients of any age with an extensive array of differential diagnoses. The primary differential considerations for ulceroglandular tularemia are inflammatory causes, including reactive lymphadenopathy, bacterial, viral, granulomatous, and mycobacterial lymphadenitis. The most similarly presenting infectious diseases are streptococcal, staphylococcal infections, cat scratch disease, mycobacterial infections, toxoplasmosis, syphilis, veneric granuloma, anthrax, Lyme disease, bubonic plague, or infectious mononucleosis [50]. The differentiation between these entities without further investigations is complex, and treatment is distinct for each pathology.

Ulceration on the skin near a neck mass can indicate an external causing agent. Nevertheless, other inflammatory causes, such as congenital and neoplastic lesions, should also be considered. Conditions mimicking tularemia-associated lymphadenitis include metastatic tumours such as squamous cell carcinoma, undifferentiated carcinoma, or metastatic disease. These can be suspected, especially in older men with smoking or alcohol abuse. Lymphoma presents similarly in younger patients, raising suspicion in a patient with night sweats and other B-symptoms. Much more extensive neck dissection and possible adjuvant radio-, immuno-, or chemotherapy present much different management of neoplastic causes than tularemia. Thus, the distinction is fundamental. Other possible differentials of cervical lymphadenopathy include benign neoplastic lesions such as pleomorphic adenoma, lipoma, schwannoma, ectopic thyroid, and Castleman’s disease [27,76]. Castleman’s disease is a non-clonal lymphoproliferative disorder presenting as non-neoplastic lymphadenopathy [79]. Additionally, inflamed congenital malformations such as branchial cleft cyst, thyroglossal duct cyst, dermoid cyst, teratoma, or vascular anomalies can present similarly to tularemia [80].

Cytopathological examination performed on lymph node fine-needle aspiration biopsy sample in tularemic lymphadenitis reveals reticulohistiocytic abscess-forming lymphadenitis. This is similar to infections with *Bartonella henselae* causing cat-scratch disease, *Yersinia pestis* causing bubonic plague, and *Chlamydia trachomatis* causing Lymphogranuloma venereum [16,50]. Granuloma with central abscess and necrosis is most commonly revealed in histopathology after lymph node excision. Necrotic granuloma formation is also seen in cases of granulomatous lymphadenitis in association with autoimmune diseases, such as rheumatoid arthritis, lupus erythematosus, or cytotoxic effects of chemoradiotherapy. Therefore, these entities should be considered, especially if no clues of the causative organism by microbiologic investigations are identified [41,81].

## 10. Treatment of Tularemia

The overall mortality of an untreated tularemia infection is 5–15% [50]. The prevailing connection to a fatal outcome is in the pulmonary form of the disease. Nevertheless, other forms may lead to terminal consequences, too. Timely diagnosis with specific antibiotic treatment significantly lowers life-threatening complications, often leaving patients entirely symptom-free. In the pre-antibiotic area, around 30% of patients with tularemia died in the USA. In infection with the more pathogenic type A, nowadays, even with prompt and appropriate antibiotic treatment, the mortality of the disease caused by this subtype is 4%. In Europe, with the less virulent subtype prevailing, however, death following tularemia is much less frequently encountered [13,27].

The antibiotic of choice depends mainly on the severity of the disease and the patient’s age (Table 1). The best minimal inhibitory concentration is streptomycin or gentamycin when taken orally for 7–14 days [28]. Ciprofloxacin, doxycycline, and chloramphenicol are also effective and should be administered for at least 14 days. Antibiotic testing of *F. tularensis* is not routinely performed in a clinical setting as antibiotic resistance of the bacterium has never been reported [82]. Thus, aminoglycosides streptomycin, gentamicin, fluoroquinolone ciprofloxacin, and tetracycline antibiotic doxycycline are most commonly used in tularemia. First-line drugs for mild to moderate tularemia cases, which predominate in Europe, are ciprofloxacin and doxycycline. Gentamicin has been reported to result in higher relapse rates, though it is often used in patients with systemic diseases and children. In younger patients, fluoroquinolones are contraindicated owing to possible damage to the muscles and skeletal system. Ciprofloxacin is a safer choice for children 1–10 years. Streptomycin or chloramphenicol are the antibiotics of choice in pregnant women with milder forms of the disease. However, gentamicin should be used in pregnant women with severe tularaemia. Possible side effects of the medication should be weighed against the severity of the disease [5,28].

In milder forms of the disease, the recommendations are to opt for treatment with ciprofloxacin or doxycycline. While fluoroquinolones such as ciprofloxacin have been reported to have a lower relapse rate (5–10%, in contrast to 10–15% in tetracyclines), several factors contribute to choosing doxycycline in cases of the disease following a tick-bite [73]. Doxycycline empirically treats other possible tick-related illnesses (e.g., Lyme disease), which is useful in the setting of a tick bite, especially before final microbiological investigations. Additionally, doxycycline is associated with fewer side effects than ciprofloxacin. In 2018, the European Medicines Agency finalised a review of severe, disabling and potentially permanent side effects of quinolone and fluoroquinolone antibiotics, endorsing new recommendations to restrict further fluoroquinolone use. Physicians are instructed to use other antimicrobials if possible. The side effects of ciprofloxacin have proven to be long-lasting, disabling, and potentially permanent, involving tendons, muscles, joints, and the nervous system [83].

Nevertheless, doxycycline is contraindicated in children and pregnancy owing to teeth discolouration and impairment of foetal bone growth [84]. The recommended duration of treatment with tetracyclines varies in the literature. According to Maurin et al. (2016), the suggested regime for doxycycline in tularemia is three weeks long to avoid disease relapses [13]. The WHO guidelines from 2007 recommend a daily dose of 200 mg of doxycycline for at least 15 days [28].

In lymphadenitis cases, surgical drainage of a colliquative lymph node is commonly indicated besides specific antibiotic treatment. However, in the experience of Atmaca et al. (2008), as reported in their study, complete surgical removal of tularemia-affected lymph nodes is often challenging, with adherent tissue preventing the dissection from the surrounding tissues [61]. Hence, as with other inflammatory swellings in the neck, tularemia-associated-lymphadenitis should be managed by a staged therapeutic approach. Therefore, the first point of reference for physicians in planning the surgical treatment of lymphadenopathy is for the lymph nodes in the neck area to be greater than 1 cm or 1.5 cm in the jugulodigastric nodes [85]. The decision to excise or drain the affected lymph node relies on colliquation in the lymph nodes, cellulitis over the skin, or sepsis. In those cases, immediate surgical drainage is required. Thus, conservative treatment with antibiotics is sufficient in the absence of liquefaction in the lymph nodes [86]. Nevertheless, even in lymphadenopathy cases without colliquation, surgical intervention in tularemia is usually performed to acquire tissue and pus samples to confirm a diagnosis and warrant antibiotic treatment.

## 11. Case Example

### 11.1. Patient History

A 71-year-old male patient from the north-western part of Slovenia with controlled arterial hypertension was referred to the Department of Otorhinolaryngology and Cervicofacial Surgery, University Medical Centre Ljubljana, Slovenia, in May 2022 because of persistent unilateral cervical lymphadenopathy. His medical history was significant for a tick bite on the skin of the neck overlying the enlarged lymph nodes approximately three weeks before admission. Shortly after the bite, a localised swelling occurred at the site of the tick bite. Initially, the swelling was located on the right side of the neck, which gradually spread caudally along the neck. The patient experienced no pain in the affected area. Fever, malaise, and any symptoms regarding respiratory and gastrointestinal tract involvement were absent throughout the disease. The patient first sought treatment at the regional secondary outpatient clinic approximately ten days after the tick bite. He was examined and empirically prescribed with oral antibiotic amoxicillin/clavulanic acid. After ten days of treatment, no symptom resolution or improvement was reported. Ultrasound imaging of the neck a day before the referral to our department revealed a 5 cm × 1 cm × 2 cm large abscess-forming lymph node conglomerate with colliquative necrosis and supraclavicular lymph node enlargement.

The patient was otherwise healthy; immunocompetent; and without allergies or chronic, familial, and genetic illnesses. He was previously treated for arterial hypertension and haemorrhoids, but had no regular medication prescribed at the time of admission. He was a non-smoker, retired from work, an amateur rabbit breeder, and often spent time outdoors hunting. He previously received vaccinations against tick-borne meningoencephalitis and COVID-19. In the past, he underwent surgical treatment for an inguinal hernia and tonsillectomy.

### 11.2. Clinical Examination

**Figure 2:**
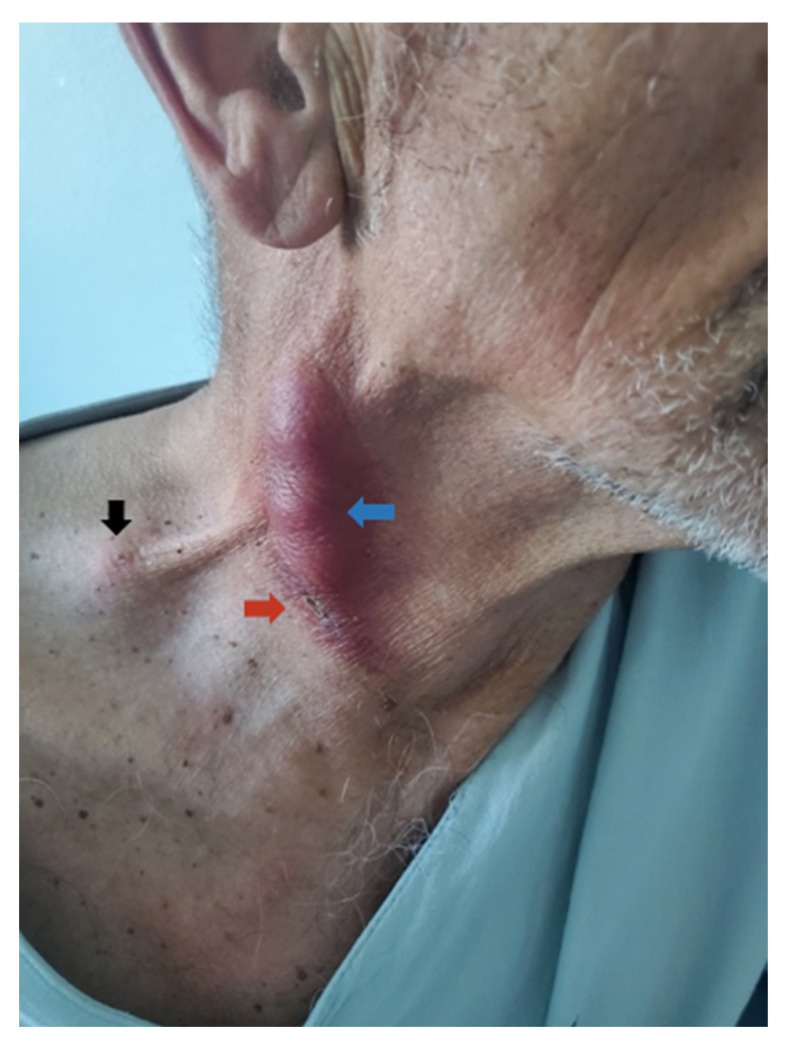
Cervical lymphadenopathy in a patient with ulceroglandular tularemia. A fluctuating neck mass is seen over the right sternocleidomastoid muscle (blue arrow), a superficial scab near the neck mass—a possible tick bite location and bacteria entry site (red arrow)—and enlarged supraclavicular lymph nodes on the right side (black arrow). Blue and red arrows anatomically correspond to Figure 1A–C and a black arrow to Figure 1D.

Upon admission to our department, a mature crust with surrounding skin erythema and two palpable subcutaneous fluctuating masses, each measuring approximately 2.5 cm in diameter, were identified (Figure 2). These findings were distributed along the superficial cervical lymph nodes overlying the right sternocleidomastoid muscle. A solid round ipsilateral supraclavicular lymph node 2 cm large in diameter was also described. In addition, post-tonsillectomy scars were observed in the oropharynx. The rest of the otorhinolaryngological examination findings were unremarkable.

### 11.3. Imaging Studies

Neck ultrasonography revealed inflammatory changes in subcutaneous fat above the right sternocleidomastoid muscle with abnormal adherent, partly colliquated lymph nodes forming a conglomerate and a conglomerate of abnormal supraclavicular lymph nodes (Figure 1).

### 11.4. Laboratory Tests Results and Infectious Disease Specialist Consultation

Laboratory testing showed an elevated sedimentation rate (52 mm/h). Other measured parameters were in the normal range. An on-call infectious diseases specialist was consulted. Ulceroglandular tularemia was proposed as a possible diagnosis owing to suggestive patient history and the colliquation in the affected lymph nodes, as observed in ultrasonography.

### 11.5. Surgical Treatment

Urgent drainage of the abscess with excision of two lymph nodes was performed under local anaesthesia the following day, considering tularemia safety precautions (i.e., FFP2 masks). Incision wounds were left open for 1 cm to aid drainage.

### 11.6. Biopsy, Histopathology, and Microbiology Findings

Lymph node tissue was sent to histopathology, which described non-specific findings—necrotising partly suppurative granulomatous inflammation.

Intraoperative and blood samples were obtained and sent to the microbiological laboratory for molecular and serological diagnostics of tularemia. DNA was extracted from the punctate of the lymph node and the pus obtained during the surgery with DNA MiniKit (Qiagen, Germany), following the manufacturer’s instructions. Tularemia was confirmed with real-time PCR targeting the *ISFtu2* region [87] and nested-PCR targeting the *F. tularensis FopA* gene [88]. Subsequent sequencing analysis of *FopA* gene confirmed *F. tularensis* subspecies *holarctica* (type B). In the biosafety level 3 laboratory, we inoculated the lymph node punctate on enriched chocolate agar and incubated for ten days, but the cultivation was unsuccessful. Additionally, serology was performed on the serum sample taken on the day of admission, three weeks after the tick bite. Indirect immunofluorescence assay (Francisella/Brucella 12-well MIF Substrate Slides; Fuller Laboratories, CA, USA) confirmed specific antibodies against *F. tularensis*: IgG > 1:1024 and IgM > 1:1024. Testing for tuberculosis was negative.

### 11.7. On-Ward Management

After microbiological confirmation of diagnosis, a two-week treatment with doxycycline was initiated according to additional infectious disease specialist consultation. Following tularemia safety guidelines, the patient did not need to be isolated. However, standard on-ward safety precautions were adopted. The patient was discharged from the hospital the day after admission.

### 11.8. Follow-Up and Outcome

Subsequent follow-up was to be held at the infectious disease outpatient clinic. After the completed antibiotic treatment, the patient was advised to make another appointment at our department in case of persisting or worsening supraclavicular lymph node enlargement. The first check-up at the infectious disease specialist was 15 days after discharge; the patient presented in good health, and the excision site on the neck was healed, showing no signs of infection, swelling, or fluctuation. The two-week doxycycline treatment regimen was completed. The previously described supraclavicular lymph node was seen with its size unchanged. Careful observation was advised, with a follow-up ultrasound examination in the case of oedema, erythema, or pain over the lymph node area.

## 12. Case Report Discussion

The sporadic case of ulceroglandular tularemia in our patient in many aspects fits the described characteristics of this disease and its management according to the reviewed literature. First, the patient presented with the most common form of the disease, showing skin ulceration, which developed into localized lymphadenopathy on the site of a tick bite after a brief inoculation period. These findings are similar to those reported in the literature. Conversely to the classic presenting clinical picture, though, the patient experienced no systemic symptoms, such as fever or myalgia. Likewise, the patient had no other otorhinolaryngological complaints. This stresses the importance of considering this disease even without obvious clues of infectious diseases.

In our patient, the diagnosis of tularemia was established similarly as suggested in the literature. Based on our patient’s age, epidemiologic information, clinical examination, and neck ultrasonography, the initial intention was to exclude other inflammatory causes of lymphadenitis. By failure of symptom improvement by previously administered beta-lactam antibiotics, the common staphylococcal and streptococcal infections were promptly excluded upon referral. Tularemia was first suspected by infectious disease specialist consultation as a possible cause of neck lymph node colliquation and necrosis findings seen upon sonography. These are typical for tularemia-associated lymphadenitis, according to the findings in the literature. Ultrasonography was, therefore, crucial for the initial evaluation of the neck mass, consistent with the reviewed articles. Owing to the patient’s tick-bite history and the high incidence of borreliosis in our geographical location, borrelial lymphocytoma was also considered. Our patient’s final diagnosis after a referral was confirmed after obtaining samples by intraoperative lymph node drainage using real-time PCR and serology. According to the literature, these are the most commonly used and reliable methods to confirm tularemia infections. Compliant with the instructions of the consulted infectious disease specialist, PCR results, serology, and clinical presentation were sufficient for establishing the diagnosis. In the biosafety level 3 laboratory, we inoculated the lymph node punctate on enriched chocolate agar and incubated it for ten days, but the cultivation was unsuccessful.

In our patient, the diagnosis was delayed for around three weeks after the onset of clinical symptoms, which could be attributed to the fact that the patient experienced no symptoms other than confined swelling on the neck over the wound of the previously inflicted tick bite. The absence of pain or severe other quality-of-life-impairing symptoms could be the reason the patient is seeking treatment only ten days after the onset of symptoms. The primary care physician initially prescribed beta-lactam antibiotics, prolonging the time to diagnosis for another ten days, a common diagnosis-limiting factor according to the literature. The relatively short delay in specific treatment contributed to the lymph node suppuration. In addition, it probably contributed to the fact that the less affected supraclavicular lymph nodes, which were not surgically treated, remained enlarged even after antibiotic treatment.

Nevertheless, life-impairing and damaging complications of neck lymph node infections were prevented in our patient by timely surgical drainage of the colliquated lymph nodes and targeted antibiotic treatment. The antibiotic of choice was 100 mg of doxycycline twice daily for 14 days, which is the most appropriate in a patient with a history of a tick bite, according to the reviewed literature. However, the decision of the infectious disease specialist consultation in our case for a two-week antibiotic treatment was made because the patient presented with a mild form of the disease without any systemic symptoms. Our case, therefore, demonstrates the importance of reconsidering the necessity of a prolonged systemic antibiotic course in a patient without systemic symptoms and successful surgical management of localised tularemia.

## 13. Conclusions

Tularemia should be suspected as a possible diagnosis in patients with tick-bite-associated cervical lymphadenopathy, especially in endemic areas. Even though cervical ulceroglandular tularemia is more commonly described in children, adults with similar complaints should not be disregarded. With the increase in sporadic cases and outbreaks of tularemia in Europe in the past decades, it is crucial to consider *F. tularensis* as a pathogen causing cervical lymphadenopathy to avoid misdiagnosis and mistreatment. The diagnosis is most commonly confirmed by serology and/or PCR amplification of bacterial DNA obtained in tissue samples. Imaging with ultrasound is vital in an initial assessment of the fluctuating neck mass to aid diagnosis and direct the extent of further management. The treatment involves oral or intravenous antibiotics and surgical drainage of the affected colliquated lymph nodes. As in our case, adequate and prompt treatment prevents local and systemic complications.

## Figures and Tables

**Table 1:** Recommended treatment for tularemia.

Disease Severity	Treatment Regimen
Severe to moderate infection	● Streptomycin 7.5 mg–1 g IM or IV, twice daily, 7–10 days OR
	● Gentamicin or tobramycin 5 mg/kg IV, once or twice daily, 10 days
	*Children:*
	● Gentamicin 2.5 mg/kg IV, three times daily, with OR without ciprofloxacin in 10–15 mg/kg orally, twice daily
Mild infection	● Ciprofloxacin 400 mg IV or 750 mg orally, twice a day, 14–21 days OR
	● Doxycycline 100 mg orally or IV, twice a day, 14–21 days
	*Children:*
	● above 8 years old: doxycycline 2.2 mg/kg orally, twice daily
	● 1–10 years old: ciprofloxacin 10–15 mg/kg orally, twice daily
Hematogenous meningitis	● Aminoglycoside + chloramphenicol 50–100 mg/kg/day IV in 4 divided doses
Pregnancy	● Streptomycin or chloramphenicol 15 mg/kg, four times a day, 14 days
Prophylaxis for aerosol exposure	● Doxycycline 100 mg orally, twice daily, 14 days OR
	● Ciprofloxacin 500 mg, orally, twice daily, 14 days

Based on WHO guidelines and reproduced with permission from Max Maurin and Miklós Gyuranecz, The Lancet Infectious Diseases; published by Elsevier, 2016 (license number: 5367520082599) [13,28] IV—intravenously, IM—intramuscularly.

## Data Availability

Not applicable.
